# Systematic screening versus clinical gestalt in the diagnosis of pulmonary embolism in COVID-19 patients in the emergency department

**DOI:** 10.1371/journal.pone.0283459

**Published:** 2023-03-23

**Authors:** Inge H. Y. Luu, Tim Frijns, Jacqueline Buijs, Jasenko Krdzalic, Martijn D. de Kruif, Guy J. M. Mostard, Hugo ten Cate, Remy J. H. Martens, Remy L. M. Mostard, Math P. G. Leers, Daan J. L. van Twist

**Affiliations:** 1 Department of Internal Medicine, Zuyderland Medical Centre, Sittard/Heerlen, The Netherlands; 2 Department of Radiology, Zuyderland Medical Centre, Sittard/Heerlen, The Netherlands; 3 Department of Pulmonology, Zuyderland Medical Centre, Sittard/Heerlen, The Netherlands; 4 Department of Internal Medicine and Biochemistry, Cardiovascular Research Institute Maastricht (CARIM), Maastricht University Medical Centre, Maastricht, The Netherlands; 5 Department of Clinical Chemistry and Haematology, Zuyderland Medical Centre, Sittard/Heerlen, The Netherlands; Istanbul University-Cerrahpasa, Cerrahpasa Medical Faculty, TURKEY

## Abstract

**Background:**

Diagnosing concomitant pulmonary embolism (PE) in COVID-19 patients remains challenging. As such, PE may be overlooked. We compared the diagnostic yield of systematic PE-screening based on the YEARS-algorithm to PE-screening based on clinical gestalt in emergency department (ED) patients with COVID-19.

**Methods:**

We included all ED patients who were admitted because of COVID-19 between March 2020 and February 2021. Patients already receiving anticoagulant treatment were excluded. Up to April 7, 2020, the decision to perform CT-pulmonary angiography (CTPA) was based on physician’s clinical gestalt (clinical gestalt cohort). From April 7 onwards, systematic PE-screening was performed by CTPA if D-dimer level was ≥1000 ug/L, or ≥500 ug/L in case of ≥1 YEARS-item (systematic screening cohort).

**Results:**

1095 ED patients with COVID-19 were admitted. After applying exclusion criteria, 289 were included in the clinical gestalt and 574 in the systematic screening cohort. The number of PE diagnoses was significantly higher in the systematic screening cohort compared to the clinical gestalt cohort: 8.2% vs. 1.0% (3/289 vs. 47/574; p<0.001), even after adjustment for differences in patient characteristics (adjusted OR 8.45 (95%CI 2.61–27.42, p<0.001) for PE diagnosis). In multivariate analysis, D-dimer (OR 1.09 per 1000 μg/L increase, 95%CI 1.06–1.13, p<0.001) and CRP >100 mg/L (OR 2.78, 95%CI 1.37–5.66, p = 0.005) were independently associated with PE.

**Conclusion:**

In ED patients with COVID-19, the number of PE diagnosis was significantly higher in the cohort that underwent systematic PE screening based on the YEARS-algorithm in comparison with the clinical gestalt cohort, with a number needed to test of 7.1 CTPAs to detect one PE.

## Introduction

Pulmonary embolism (PE) appears to be a frequent complication in patients with COVID-19 [1–3]. Early diagnosis and treatment of PE in these patients is vital as untreated PE may be life-threatening [4]. Unfortunately, diagnosing concomitant PE in patients with COVID-19 remains challenging, as signs, symptoms, and biomarkers of PE and COVID-19 show a wide overlap. Consequently, deciding on diagnostic testing for concurrent PE based on the intuitive clinical gestalt of physicians may result in underdiagnosis of PE in these patients.

In the first COVID-19 wave, alarming reports on the high incidence of PE in COVID-19 patients in the intensive care unit [5] prompted our large teaching hospital to introduce systematic screening for PE, based on the YEARS-algorithm [6], in all patients in the Emergency Department (ED) who were admitted for hospital care because of (suspected) COVID-19 [7].

In this study, we aimed to 1) compare the diagnostic yield of PE screening in ED patients with COVID-19 before and after implementation of the systematic screening protocol, 2) identify risk factors associated with PE in COVID-19 patients upon admission, and 3) evaluate differences in the number of PE diagnoses over time.

## Methods

We included all ED patients aged 18 years and over who were admitted to Zuyderland Medical Centre, the Netherlands, between March 1, 2020 and February 28, 2021, with a definite diagnosis of COVID-19. A definite diagnosis of COVID-19 was defined as having clinical symptoms suspected for COVID-19 (according to the WHO case definition for suspected COVID-19) [8] and either a positive reverse transcriptase-polymerase chain reaction (RT-PCR) for SARS-CoV-2 upon ED presentation, a COVID-19 CT-classification score (CO-RADS) [9] 4 or 5 on chest computed tomography (CT) obtained at the ED, and/or a pending test result for SARS-CoV-2 upon ED presentation that was subsequently confirmed by a positive RT-PCR during hospitalization.

Data collection included patient demographics, clinical manifestations, laboratory data, and radiographic characteristics. The Charlson Comorbidity Index (CCI) was calculated for each patient [10]. All data were registered in the ZuydErLand COVID-19 regiStry (ELVIS).

Patients were excluded from the present analysis if they were on therapeutic anticoagulation therapy (low-molecular-weight heparin, vitamin-K-antagonists, or direct oral anticoagulants), or had a contraindication to CT-pulmonary angiography (CTPA; e.g., allergy to intravenous contrast agents, impaired renal function, or inability to cooperate) or to anticoagulant treatment (e.g., major bleeding). Patients with acute respiratory failure who were not able to undergo CTPA during ED stay as they required immediate high-flow oxygen therapy or mechanical ventilation were excluded as well. Patients in whom the diagnostic protocol was violated (e.g., no D-dimer testing during ED presentation or no CTPA despite indicated according to the protocol) were also excluded from the present analysis.

### Systematic screening protocol for PE

In the beginning of the COVID-19 pandemic (from March 1 through April 6, 2020), imaging studies in ED patients consisted of chest X-ray and/or non-contrast-enhanced CT of the chest to support diagnosis of COVID-19. CTPA was only performed in case of clinical suspicion for PE, based on the “intuitive” clinical gestalt of the attending physician, who built the decision on the medical history, physical examination, and laboratory results. D-dimer was only measured if specifically ordered by the physician. Hereafter, this cohort will be referred to as the ‘clinical gestalt cohort’.

In response to the worrying reports that COVID-19 patients might be at risk for developing PE [11, 12], the local clinical protocol changed. From April 7, 2020 onwards, systematic screening for PE, based on the YEARS-algorithm [6], was implemented for all ED patients with suspected or confirmed COVID-19 who were subsequently admitted to the hospital (hereafter, the ‘systematic screening cohort’). The YEARS-algorithm applies a clinical pretest probability to identify patients in whom a higher D-dimer threshold can be used to safely rule out PE without performing CTPA and consists of three clinical items: 1) clinical signs of deep vein thrombosis, 2) haemoptysis, and 3) PE as the most likely diagnosis. In case of ≥1 YEARS-items, a D-dimer cut-off level of ≥500 ug/L is applied. If none of the three YEARS-items are present, the cut-off is increased to 1000 ug/L. Patients who had D-dimer values below the cut-off were considered to have PE excluded and underwent a non-contrast-enhanced chest CT or a chest X-ray as part of the standard clinical protocol. In patients with D-dimer values above the cut-off value, CTPA was performed. The YEARS-algorithm is externally validated and has been endorsed in guidelines for the diagnostic work-up for PE [13, 14]. It has previously been shown that the YEARS-algorithm can safely be used in ED patients [15], and possibly even in COVID-19 setting [16].

D-dimer levels were measured using CS5100 automated blood coagulation analysers (Sysmex Corporation, Kobe, Japan; upper reporting limit 35000 μg/L). CT examinations were performed using 64-detector-row CT-scanners (Incisive, Philips Medical Systems, The Netherlands, and Definition Flash, Siemens Healthineers, Germany) and evaluated by board-certified radiologists. The full CTPA protocol is provided in S1 Appendix.

### Ethics

This study was approved by the Ethics Committee of Zuyderland Medical Centre (METCZ20200076). All COVID-19 patients admitted to Zuyderland MC were registered in the ZuydErLand COVID-19 regiStry (ELVIS). All patients received written information about the registry and an opt-out form in case they did not want to participate. This opt-out mechanism was approved by the Ethics Committee of Zuyderland Medical Centre. None of the patients included in this study objected to participation.

### Statistical analysis

Differences between two groups were evaluated with the Chi-square test or Fisher’s exact test for categorical data and the Student’s t-tests or Mann-Whitney U-tests for continuous variables. Continuous variables were reported as means with standard deviation (SD) for parametric data and median with interquartile range (IQR) for non-parametric data. For the baseline characteristics, cases with missing data were removed from the denominator when calculating percentages. The number needed to test (NNT), defined as the number of people that need to undergo a CTPA to detect one PE, was calculated as the inverse of PE prevalence among patients who underwent CTPA. PE rates in the clinical gestalt and systematic screening cohorts, and among the three periods within the systematic screening cohort (April to July 2020, August to November 2020, and December 2020 to February 2021) were compared using bivariate analysis and multivariable logistic regression analysis. To identify factors associated with PE, univariate and multivariate regression analyses were performed on the data of the systematic screening cohort. Odds ratios (ORs) with 95% confidence intervals (CIs) were calculated for a comparison between the groups with or without PE for categorical variables. Associations with p<0.10 in the univariate logistic regression models and those considered biologically plausible were included in the multivariate logistic regression model. Values of p<0.05 were considered statistically significant. All analyses were performed using SPSS software (version 28.0; IBM Corp., Armonk, NY, USA).

## Results

In the one-year study period, a total of 1095 patients with a definite diagnosis of COVID-19 were admitted to the hospital via our ED. Of these, 168 were excluded because they used therapeutic dose anticoagulant drugs.

**Fig 1:**
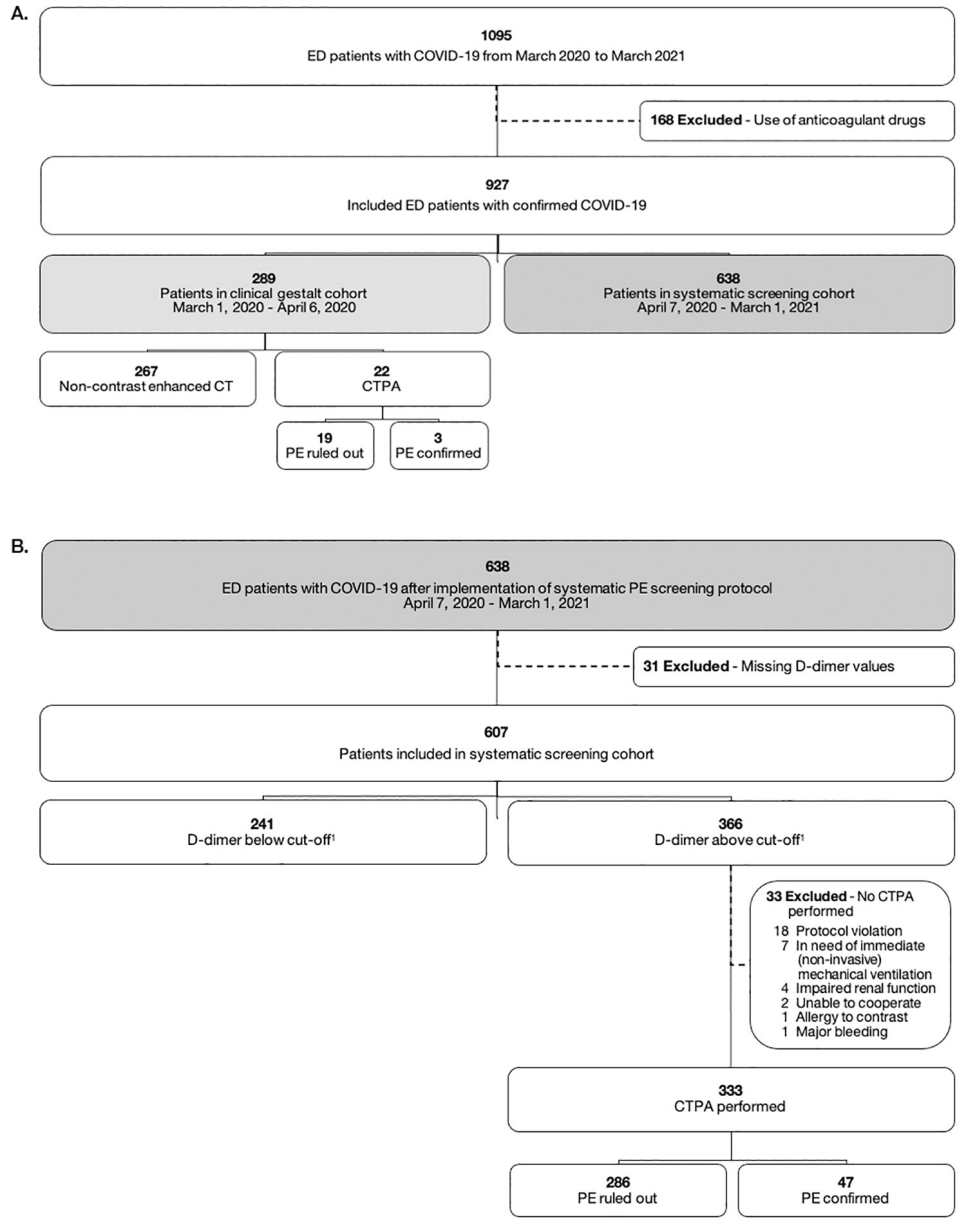
Flowchart of study population, showing (A) cohorts and (B) further details of the systematic screening cohort. ^1^D-dimer cut-off ≥ 500 ug/L if ≥ 1 YEARS items; D-dimer cut-off is increased to 1000 ug/L if 0 YEARS items. ED, emergency department; CTPA; computed tomography pulmonary angiogram; PE, pulmonary embolism.

Of the remaining 927 patients, 289 were included in the clinical gestalt cohort from March 1 to April 6, 2020 and 638 patients in the systematic screening cohort from April 7, 2020 to February 28, 2021 (Fig 1A).

In the systematic screening cohort, 31 patients (4.9%) did not undergo D-dimer testing and were therefore excluded. Following the YEARS-algorithm, CTPA was indicated in 366 of the 607 patients (60.3%) (Fig 1B). Eight patients with contraindications to CTPA were excluded. Another 7 patients with acute respiratory failure could not undergo CTPA upon admission as they required immediate high flow oxygen therapy or mechanical ventilation. Protocol violation occurred in 18 patients in whom CTPA was not performed because the attending physician overruled the protocol as he/she considered the clinical suspicion of PE negligible (n = 6) or because of unknown reasons (n = 12). Thus, 333 patients underwent CTPA according to the YEARS-algorithm. Including the 241 patients in whom CTPA was not indicated based on the YEARS-algorithm, a total of 574 patients followed the prespecified protocol and were included in the systematic screening cohort.

### Patient characteristics

Characteristics of the study population are shown in Table 1. Patients were predominantly male (63.8%) and on average 67.6±13.2 years old. Patients in the systematic screening cohort more often had diabetes mellitus (19.0% vs. 25.4%, p = 0.04), but the prevalence of other comorbidities did not differ significantly.

**Table 1:** Baseline characteristics.

Variables	Clinical gestalt n = 289	Systematic screening n = 574	*P*-value
**Demographics**			
Age (year)	68.2 ± 12.7	67.3 ± 13.4	0.359
Male sex, *n* (%)	186 (64.4)	365 (63.6)	0.824
BMI (kg/m^2^)	28.8 ± 5.5	28.4 ± 5.8	0.395
> 30 kg/m^2^, *n* (%)	85 (32.4)	167 (31.0)	0.689
**Comorbidity**			
Hypertension, *n* (%)	108 (37.4)	204 (35.5)	0.597
Diabetes mellitus, *n* (%)	55 (19.0)	146 (25.4)	0.036
COPD, *n* (%)	42 (14.5)	84 (14.6)	0.968
Asthma, *n* (%)	32 (11.1)	49 (8.5)	0.228
Malignant neoplasms, *n* (%)	9 (3.1)	31 (5.4)	0.132
Hematological malignancies, *n* (%)	6 (2.1)	11 (1.9)	0.873
Cardiovascular disease, *n* (%)	60 (20.8)	124 (21.6)	0.776
Chronic renal insufficiency, *n* (%)	29 (10.0)	51 (8.9)	0.583
Cerebrovascular disease, *n* (%)	28 (9.7)	74 (12.9)	0.169
Charlson Comorbidity Index	3 [2–5]	3 [2–5]	0.602
**Time symptom onset to presentation ED (days)**	8.6 ± 5.7	7.8 ± 6.0	0.076
**Vital signs**			
Heart rate (bpm)	93 ± 19	92 ± 18	0.169
Mean arterial pressure (mmHg)	98 ± 13	98 ± 14	0.790
Temperature (°C)	38.1 ±1.1	38.0 ± 1.1	0.203
**Laboratory findings**			
Hemoglobin (mmol/L)	8.7 [7.9–9.4]	8.5 [7.7–9.2]	0.032
Leukocytes (×10E9/L)	6.6 [5.1–9.1]	7.2 [5.2–9.7]	0.303
Platelet count, (×10E9/L)	214 [165–274]	211 [166–269]	0.667
Lymphocyte count (×10E9/L)	0.82 [0.57–1.18]	0.87 [0.60–1.20]	0.346
CRP (mg/L)	82 [45–130]	81 [40–137]	0.633
Ferritin (*μ*g/L)	759 [423–1357]	749 [352–1461]	0.825
D-dimer (*μ*g/L)	977 [559–2103]	1087 [629–2255]	0.234
Lactate (mmol/L)	1.54 [1.2–1.93]	1.50 [1.22–1.96]	0.760

Data shown as mean ± standard deviation or median [interquartile range], unless otherwise stated.

BMI, body mass index; bpm, beats per minute; COPD, chronic obstructive pulmonary disease; CRP, C-reactive protein; ED, emergency department.

### Clinical gestalt versus systematic screening

In the clinical gestalt cohort, thus before the implementation of the prespecified PE screening protocol, CTPA was performed in 22 out of 289 patients (7.6%) in whom PE was suspected by the attending physician based solely on presenting signs, symptoms, laboratory results, and physician’s clinical gestalt. D-dimer was measured in 181 patients (62.6%). Of the 22 patients who underwent CTPA, D-dimer values were measured in 19 (86.4%) and ranged from 386 μg/L to 29456 μg/L. CTPA revealed PE in 3 of these 22 patients (13.6%) (Fig 1A), all located in segmental arteries. The NNT by CTPA was 7.3. The overall prevalence of detected PE in the clinical gestalt cohort was 1.0% (3 out of 289).

In the systematic screening cohort, 333 patients (58.0%) underwent CTPA according to the YEARS-algorithm. PE was confirmed in 47 of them (14.1%) (Fig 1B), resulting in a NNT by CTPA of 7.1. The overall PE incidence in the complete systematic screening cohort, including the 241 patients in whom PE was considered to be ruled out based on the YEARS-algorithm, was 8.2% (47 out of 574). Segmental arteries were most commonly affected (n = 30, 63.8%), followed by subsegmental branches (n = 14, 29.8%), whereas central embolisms accounted for 6.4% (n = 3) of all cases.

The number of PE diagnoses was significantly higher in the systematic screening cohort compared with the clinical gestalt cohort: 8.2% vs. 1.0% (p<0.001). When adjusted for differences in age, gender, and CCI between groups, the odds ratio for the diagnosis of PE in the systematic screening cohort versus the clinical gestalt cohort was OR 8.45 (95% CI 2.61–27.42, p<0.001).

### Risk factors for PE

**Fig 2:**
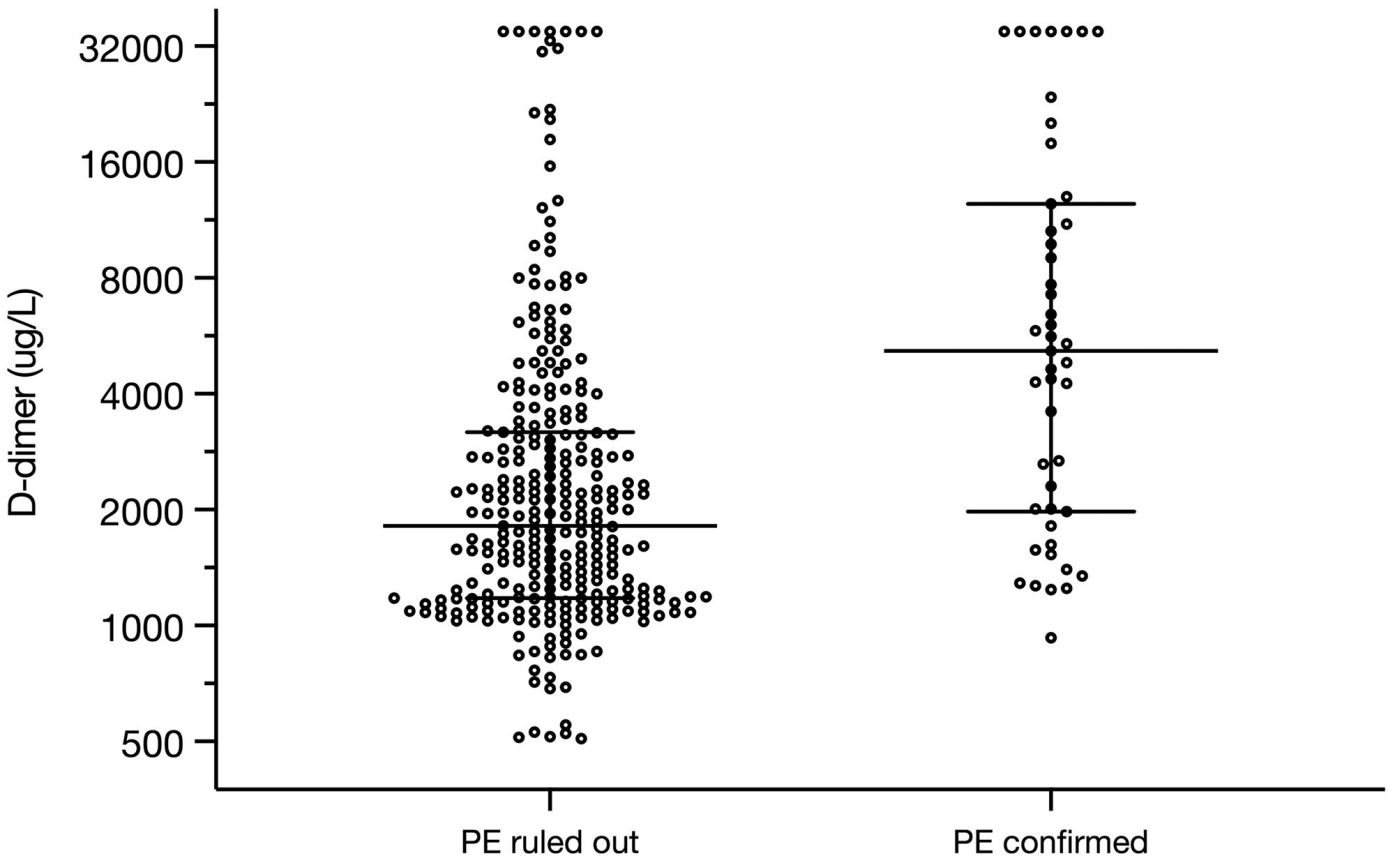
Scatter plot of the D-dimer levels in patients who underwent CTPA according to the YEARS algorithm, in whom PE was ruled out and confirmed, on a base-2 log scale. Each dot represents one patient. Horizontal lines show the median, error bars show the interquartile range. PE, pulmonary embolism.

In the group of patients who underwent CTPA, D-dimer level was significantly higher in patients with PE (median (IQR) 5175 (1977–12458) μg/L) as compared with patients without PE (1814 (1179–3178) μg/L, p<0.001) (Fig 2). The lowest D-dimer value among patients with PE was 1349 μg/L for patients who met zero YEARS-items and 932 ug/L for patients who met ≥1 YEARS-items.

In a univariate analysis of the systematic screening cohort (n = 574), significant predictors for PE included D-dimer (OR 1.10 for every 1000 μg/L increase, 95% CI 1.07–1.13, p<0.001), C-reactive protein (CRP) >100 mg/L (OR 2.61, 95% CI 1.42–4.81, p = 0.002), and lactate >1.8 mmol/L (OR 2.44, 95% CI 1.30–4.57, p = 0.006), but not age or BMI (Table 2). In multivariate analysis, however, only D-dimer (OR 1.09 for every 1000 μg/L increase, 95% CI 1.06–1.13, p<0.001) and CRP >100 mg/L (OR 2.78, 95% CI 1.37–5.66, p = 0.005) were independently associated with PE.

**Table 2:** Regression analysis for factors associated with PE in ED patients with COVID-19.

	Univariate analysis			Multivariate analysis		
Variables	OR	95% CI	*P*-value	OR	95% CI	*P*-value
**Age per 5 years**	1.08	0.96–1.21	0.20			
**Male sex**	1.01	0.54–1.88	0.97			
**BMI (kg/m** ^**2**^ **)**						
<25	Reference					
25–30	1.06	0.51–2.18	0.88			
≥30	0.69	0.30–1.59	0.38			
**Chronic comorbidity**						
COPD	0.84	0.35–2.05	0.71			
Malignant neoplasms	2.29	0.84–6.28	0.11	2.54	0.83–7.73	0.10
CCI ≥4	1.55	0.84–2.84	0.16			
**Vital signs**						
Heart rate >100 bpm	1.73	0.94–3.19	0.08	1.37	0.67–2.78	0.39
Temperature ≥38.5°C	0.56	0.27–1.16	0.12			
**Laboratory results**						
D-dimer per 1000 *μ*g/L	1.10	1.07–1.13	<0.001	1.09	1.06–1.13	<0.001
CRP >100 mg/L	2.61	1.42–4.81	0.002	2.78	1.37–5.66	0.005
Ferritin >900 *μ*g/L	1.66	0.91–3.02	0.10			
Lactate >1.8 mmol/L	2.44	1.30–4.57	0.006	1.77	0.89–3.54	0.11

BMI, body mass index; bpm, beats per minute; CCI, Charlson Comorbidity Index; CI, confidence interval; COPD, chronic obstructive pulmonary disease; CRP, C-reactive protein; OR, Odds ratio.

Other variables listed in Table 1 but not shown here were not associated with PE in univariate analysis.

Additional receiver operating characteristic (ROC) curve analyses were performed to assess the predictive value of D-dimer and CRP for PE (area under the curve (AUC) 0.858 (95% CI 0.814–0.902) and 0.637 (95% CI 0.547–0.727) respectively; S1 Fig). The combination of D-dimer and CRP (AUC 0.758 (95% CI 0.677–0.838)) did not improve the predictive ability compared to D-dimer alone.

### PE prevalence over time

**Fig 3:**
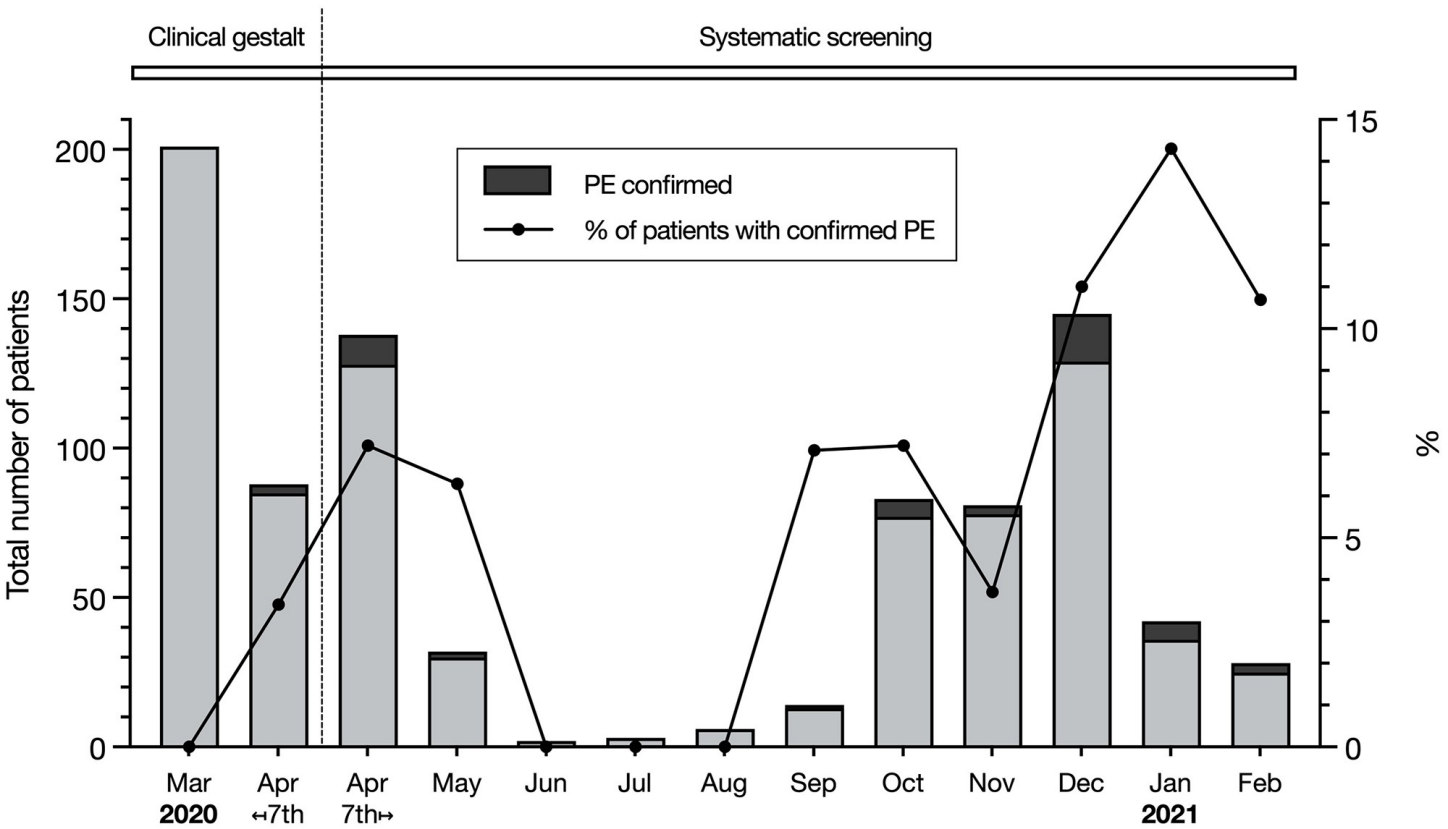
Chart of the total number of ED patients with COVID-19 in whom pulmonary embolism was confirmed (left axis, represented as the bar graph) and the proportion of confirmed PE among ED patients with COVID-19 (right axis, shown with the line graph), plotted for each month. ED, emergency department; PE, pulmonary embolism.

Fig 3 illustrates the prevalence of PE over time, plotted for each month. In the first 8 months after implementation of the systematic screening protocol, PE prevalence was relatively stable: 6.9% from April 7 to July 2020 and 5.4% from August to November 2020. Thereafter, however, PE prevalence increased to 11.6% (December 2020 to February 2021), with an OR of 2.03 (95% CI 1.11–3.71, p = 0.02) for PE diagnosis in the last three months versus previous periods. However, we also observed significant differences in patient characteristics across these three periods for age (64.5±14.5 vs. 67.2±13.6 vs. 69.7±11.9 years respectively, p<0.001), CCI (3 (1–5) vs. 3 (2–5) vs. 4 (2–5), p = 0.012), and the inflammatory markers CRP (60 (17–105) vs. 87 (52–148) vs. 89 (43–140) mg/L, p<0.001) and ferritin (558 (260–1151) vs. 818 (435–1580) vs. 864 (384–1531) μg/L, p = 0.002) (S1 Table). After adjusting for these differences, PE prevalence in the last three months was still (borderline) statistically significantly higher (OR 1.85, 95% CI 1.00–3.41, p = 0.05 vs. previous periods).

## Discussion

In this large single-centre study among COVID-19 patients in the ED, we compared the diagnostic yield of PE screening based on physician’s clinical gestalt versus systematic screening using the YEARS-algorithm. PE was diagnosed in 1.0% of the patients in the clinical gestalt cohort and 8.2% of those in the systematic screening cohort. Even after adjustment for small differences in patient characteristics, the incidence of PE was substantially higher in the systematic screening cohort, with an adjusted OR of 8.45, indicating that systematic PE screening results in a considerable number of additional PE diagnoses.

Despite this 8.5-fold increase in PE diagnoses in the systematic screening cohort, the NNT by CTPA did not differ. Although we cannot rule out a difference in the actual PE prevalence between the two cohorts, it seems highly unlikely that the chance of having PE would have increased by 8.5 times in the systematic screening cohort. Moreover, another large study that was conducted in the ED, but in which CTPA was only performed in case of suspected PE, found a low PE incidence of 0.5% in March to April 2020 [17], in line with the number PE diagnoses in our clinical gestalt cohort (1.0%). Although different cohorts may vary and cannot be compared directly, the difference in the number PE diagnoses compared to our systematic screening cohort (7% in April 2020), is very large. Hence, the increased identification of PE is presumably the result of active systematic screening that subsequently led to capturing of more true PE cases, which also suggests that a substantial number of PEs have been missed in the clinical gestalt cohort. Although physician’s clinical gestalt is generally considered to have important diagnostic value in patients with suspected PE [18, 19], the wide overlap of symptoms between COVID-19 and PE is likely to complicate the clinician’s judgement in patients with COVID-19 [20]. Therefore, we advocate the use of systematic PE screening among ED patients with COVID-19 requiring hospitalization.

The present study supports the concept of a relatively high PE prevalence in COVID-19 patients at ED presentation. Previous smaller studies on systematic screening in COVID-19 patients in the ED showed similar PE prevalence: In a small retrospective study CTPA was performed in all 106 patients and showed a PE prevalence of 14.2% [21]. In another study in 445 patients, CTPA was performed in case of D-dimer >1000 μg/L and revealed a NNT by CTPA of 6.5, with an overall PE prevalence of 5.8% [22].

As expected, we observed a clear increase in CTPA examinations performed after implementation of the systematic screening protocol. Performing CTPA indiscriminately in every COVID-19 patient to screen for PE is often difficult due to logistic constraints, and undesirable because of potential side-effects of CTPA. Therefore, clinical decision rules that identify patients who are very unlikely to have PE may reduce the number of CTPAs. A previous study found a failure rate of 1.4% of the YEARS-algorithm in COVID-19 patients [23], which is commonly considered acceptable [24]. In an attempt to further reduce the number of CTPAs, several non-systematic retrospective studies proposed to use higher D-dimer thresholds, as COVID-19-associated inflammation induces an increase in D-dimer values [25–27]. However, these suggested D-dimer thresholds of 2500–2900 μg/L, based on the highest “Youden’s index” for an optimal cut-off point, yield a relatively low sensitivity. In our cohort, adjusting the D-dimer threshold from 1000 to 2500 μg/L would have resulted in 17% missed PEs. In the present study, the highest D-dimer value that still obtained a sensitivity and negative predictive value of 100% was 1349 μg/L for patients who met zero YEARS-items and 932 ug/L for patients who met ≥1 YEARS-items. As such, we do not support raising the D-dimer threshold to predict PE risk in ED patients with COVID-19.

In this study, D-dimer and CRP were found to be the only predictive factors for PE. The association between CRP and PE may be the result of upregulation of inflammatory mediators during acute thrombosis [28]. Moreover, the interplay between proinflammatory and procoagulant factors results in increased susceptibility to thrombosis during hyperinflammation [29]. Unfortunately, the addition of CRP did not improve the negative predictive value of D-dimer for PE in order to further reduce the number of CTPAs needed.

Interestingly, a variation in PE prevalence over time was observed. Aside from the summer months where PE rates dropped markedly along with the COVID-19-related hospitalizations, the proportion of confirmed PE among ED patients with COVID-19 remained broadly consistent from April to November 2020. However, in the last three months of the study period, PE prevalence increased. In the same period, patients were older, had more comorbidities (as mirrored by a higher CCI score) and more often showed signs of hyperinflammation, which may have contributed to the higher PE prevalence. Yet, after adjustment for these differences, PE prevalence in this period was still marginally increased compared with the preceding months. Possibly, differences in referral patterns and between COVID-19 virus variants may be of influence. As this study was conducted at the moment of ED presentation, administration of thromboprophylaxis and corticosteroids did not affect our observed results, since initiation of these regimens occurred after admission to the medical wards and was not usual in outpatients with COVID-19 at that time.

This study has some limitations. First, this is an observational study in which we compared the diagnostic yield of systematic screening for PE to a historical (clinical gestalt) cohort. As such, we cannot rule out that a potential time effect (e.g., different COVID-19 variants, enhanced attention to PE over time) may have affected the results. However, the surge in PE diagnoses was evident after implementation of systematic screening and remained consistent throughout the complete systematic screening period. Since the “clinical gestalt” period (March till the begin of April 2020) and the first few months of the “systematic screening” period (from April 7, 2020 onwards) fell in the same (first) COVID-19 wave in Western Europe, caused by the Alpha-variant of COVID-19, the evolution of COVID-19 variants could not have played a large role in the difference in the number of PE diagnoses in both cohorts. Thus, we are still confident that systematic PE screening is of additional value in ED patients with COVID-19. Second, by nature of the protocol, CTPA was not performed in patients with low D-dimer in whom CTPA was not indicated according to the YEARS-algorithm, and we cannot rule out PE with certainty in these patients. However, even in COVID-19 patients, normal D-dimer levels are considered to be able to safely rule out PE without CTPA [23, 30]. Another limitation is the exclusion of patients with concomitant use of anticoagulant drugs, precluding drawing conclusions in this group. Last, patients with contraindications to CTPA or anticoagulant therapy did not undergo diagnostic imaging for PE during ED visit and were therefore excluded from our analysis. However, as this applied to only 33 patients (3.8% of the study population), we do not believe this exclusion influenced the results significantly.

## Conclusion

In ED patients with COVID-19, the number of PE diagnoses was 8.5 times higher in a cohort that underwent systematic screening for PE based on the YEARS-algorithm as compared to a historical cohort which underwent PE evaluation according to unstructured clinical gestalt. Whilst this study has limitations due to its retrospective design, this finding suggests that the number of PE diagnoses may increase substantially with systematic screening. Although the number of CTPAs increased with systematic PE screening, we consider a NNT of 7.1 scans to detect one PE reasonable given the benefits of early PE diagnosis in COVID-19 patients upon hospital admission, allowing prompt and early treatment of this potential life-threatening complication.

## Supporting information

S1 Appendix CTPA scan and injection protocol. (PDF)

S1 Table Comparison of characteristics across three periods in the systematic screening cohort. Differences between groups were tested with one-way ANOVA for continuous variables and Chi-square test for categorical variables.
(PDF)

S1 Fig ROC curve analysis showing the AUC of D-dimer, CRP, and the combined application of D-dimer and CRP for predicting pulmonary embolism. AUC, area under the curve; CRP, C-reactive protein.
(TIFF)

S1 File Dataset. (SAV)
