# Supplementary material for: Systematic screening versus clinical gestalt in the diagnosis of pulmonary embolism in COVID-19 patients in the emergency department
Source: PLoS One. 2023 Mar 23;18(3):e0283459. doi: 10.1371/journal.pone.0283459 (PMC10035852; doi:10.1371/journal.pone.0283459)
Supplement: S1 Appendix — (PDF) [file pone.0283459.s001.pdf]

S1 Appendix. CTPA scan and Injection Protocol

CT examinations were acquired using 64-detector-row CTs (Incisive, Philips Medical Systems, The Netherlands, and Definition Flash, Siemens Healthineers, Germany). All CT images were acquired in a cranio-caudal direction from the level of the lung apices to the diaphragm within a single breath hold. The scans were obtained with 100 kV, 85-122 mAs (with Care Dose on), 64×0.625 mm collimation, rotation time 0.4-0.5 s and pitch 1.0-1.2. All images were reconstructed with 1.0 mm slice thickness and 1.0 mm slice increment, and an I31f kernel applied for the soft tissue images. Multiplanar reconstructions and maximum intensity projections were used at the discretion of the interpreting radiologist. Pulmonary vessels were evaluated for the presence of a clot, including subsegmental vessels. A clot was considered to be present if a vessel is completely occluded by low-attenuation material on at least two adjacent slices. The Stellant Dual CT Injector (Medrad Europe BV, The Netherlands) was used for intravenous bolus injection. Patients received 50 ml of i.v. contrast medium (Xenetix 300, Guerbet, France) injected at a flow rate of 5.0 ml/s and followed by a 45 ml NaCl chaser bolus. Individual bolus timing was performed in order to generate an individual start delay using test bolus technique at the level of the pulmonary trunk.
