# Supplementary material for: Systematic screening versus clinical gestalt in the diagnosis of pulmonary embolism in COVID-19 patients in the emergency department
Source: PLoS One. 2023 Mar 23;18(3):e0283459. doi: 10.1371/journal.pone.0283459 (PMC10035852; doi:10.1371/journal.pone.0283459)
Supplement: S1 Table — Differences between groups were tested with one-way ANOVA for continuous variables and Chi-square test for categorical variables. (PDF) [file pone.0283459.s002.pdf]

S1 Table. Comparison of characteristics across three periods in the systematic screening cohort. Differences between groups were tested with one-way ANOVA for continuous variables and Chi-square test for categorical variables.

Variables	April 7, 2020 - July 2020 (n = 175)	August 2020 - November 2020 (n = 184)	December 2020 - February 2021 (n = 215)	*p*-value
Age (year)	64.5 ± 14.5	67.2 ± 13.6	69.7 ± 11.9	<0.001*
Male sex, *n* (%)	107 (61.1)	122 (66.3)	136 (63.3)	0.592
BMI (kg/m^2)	27.8 ± 5.7	29.2 ± 6.0	28.2 ± 5.7	0.063
Charlson Comorbidity Index	3 [1-5]	3 [2-5]	4 [2-5]	0.012*
Heart rate (bpm)	92 ± 18	92 ± 18	90 ± 18	0.440
CRP (mg/L)	60 [17-105]	87 [52-148]	89 [43-140]	<0.001*
Ferritin (µg/L)	558 [260-1151]	818 [435-1580]	864 [384-1531]	0.002*
D-dimer (µg/L)	997 [552-2649]	1157 [535-2119]	1110 [711-2253]	0.509
Lactate (mmol/L)	1.49 [1.18-1.96]	1.53 [1.27-2.01]	1.49 [1.23-1.91]	0.383

Data are presented as mean ± standard deviation or median [interquartile range], unless otherwise stated.
BMI, body mass index; bpm, beats per minute; CRP, C-reactive protein.
