# Supplementary figures and images for: Systematic screening versus clinical gestalt in the diagnosis of pulmonary embolism in COVID-19 patients in the emergency department
Source: PLoS One. 2023 Mar 23;18(3):e0283459. doi: 10.1371/journal.pone.0283459 (PMC10035852; doi:10.1371/journal.pone.0283459)

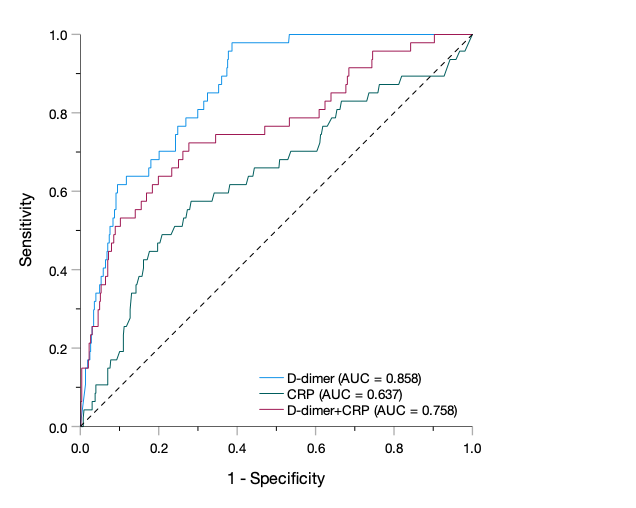

Supplement: S1 Fig — AUC, area under the curve; CRP, C-reactive protein. (TIFF) [file pone.0283459.s003.tiff]
